# Butyric acid reduced lipid deposition in immortalized chicken preadipocyte by inhibiting cell proliferation and differentiation

**DOI:** 10.1016/j.psj.2024.104171

**Published:** 2024-08-05

**Authors:** Xiaoying Liu, Kailong Qin, Chaohui Wang, Xi Sun, Yun Li, Yanli Liu, Xiaojun Yang

**Affiliations:** College of Animal Science and Technology, Northwest A&F University, Yangling, 712100, China

**Keywords:** butyric acid, differentiation, lipid deposition, ICP2, proliferation

## Abstract

The hyperplasia and hypertrophy of preadipocytes were closely related to lipid deposition in animals. Butyric acid was reported to be involved in lipid metabolism. The aim of the current study was to investigate the effect of butyric acid on the proliferation and differentiation of the immortalized chicken preadipocyte 2 (**ICP2**). ICP2 were treated respectively with 12mM butyric acid for 48h in proliferation trial and 4mM butyric acid plus 200 μM oleic acid for 3 d in differentiation trial. For the proliferation trial, RNA-seq analysis revealed that 2039 genes were significantly up-regulated and 780 genes were significantly down-regulated with 12 mM butyric acid after 48 h treatment. Concurrently, Cell cycle, DNA replication and p53 signaling pathways were down-regulated in Butyric acid group. More importantly, 12 mM butyric acid restrained the expression of cell proliferation genes such as PCNA, CDK1 and CDK2 in Butyric acid group (*P* < 0.05), and the protein expression levels of PCNA and CDK1 were also significantly decreased (*P* < 0.05). The Oil red staining revealed a fewer presence of red fat droplets in ICP2 following treatment with 4 mM butyric acid, accompanied by decreased levels of total cholesterol (**TC**) and triglycerides (TG). RNA-seq analysis shown that the number of up and down-regulated genes were 2095 and 1042 respectively in OAB group (oleic acid+butyric acid) when compared with OA group (oleic acid). Meanwhile the AMPK signaling pathway, FOXO signaling pathway and focal adhesion were significantly enriched in OAB group. Additionally, 4 mM butyric acid inhibited the expression of lipid differentiation genes including FABP4, C/EBPα, PPARγ and LPL in OAB group (*P* < 0.05), as well as lipogenesis proteins such as FABP4, C/EBP-α and PPARγ (*P* < 0.05). In conclusion, 12 mM butyric acid effectively inhibited the proliferation of ICP2 by slowing down cell cycle progression, while 4 mM butyric acid alleviated lipid deposition by reducing the production of lipid droplets through inhibiting the expression of lipid differentiation marker genes and proteins.

## INTRODUCTION

Broiler chickens were extensively raised for their substantial economic value. Nevertheless, the elevated abdominal fat percentage presented a considerable obstacle to their growth, as well as impacting feed conversion rate (Moreira et al., 2018). Consequently, addressing excessive fat deposition was a pressing issue in the broiler industry. Fat deposition was a process influenced by numerous factors. At the cellular level, fat synthesis hinges on the augmentation of both number and size in adipocyte, which were closely related to the proliferation and differentiation of adipocyte (Nematbakhsh et al., 2021; Park et al., 2021), as well as the synthesis and deposition of triglyceride (Zhang et al., 2015). Therefore, regulating the proliferation and differentiation of adipocytes as the vital factor in relieving fat deposition in broiler chickens.

Short chain fatty acids (**SCFA**) were important glycolipid compounds that play a crucial role in regulating the energy balance and metabolism in animals (Cronin et al., 2021; Wang et al., 2020). Butyric acid was a member of SCFA, which could influence the expression of numerous genes and proteins associated with adipocytes proliferation and differentiation, thereby regulate animal lipid metabolism (Coppola et al., 2021). Numerous studies have demonstrated the beneficial effects of butyric acid in mitigating obesity induced by high fat dietary (Arnoldussen et al., 2017; Fang et al., 2019). It was reported that 3 mM and 10 mM butyric acid inhibited insulin-stimulated de novo lipogenesis in primary rat adipocytes (Heimann et al., 2015). However, maternal dietary addition of 1% sodium butyrate increased intramuscular fat deposition in offspring mice (Huang et al., 2017). The previous study revealed that 0.5 µM sodium butyrate inhibited the lipogenic differentiation of human mesenchymal stem cells (Chen et al., 2007). These studies indicated that the function of butyric acid vary depending on the dosage in different experimental models.

In summary, extensive research predominantly used mammalian models to explore the impact of butyric acid on lipid metabolism. Considering the distinct anatomical and lipid metabolism characteristics of broiler chickens, butyric acid might exert diverse regulatory effects on fat deposition in broiler chickens compared to mammals. Despite evidence demonstrated that dietary addition with 0.1% sodium butyrate inhibited fat deposition in the liver and abdominal fat in broilers (Zhao et al., 2022), but underlying mechanism remained incompletely elucidated. Therefore, immortalized chicken preadipocyte 2 (**ICP2**) were used in the current study to explore whether butyric acid could affect the proliferation and differentiation, aiming to provide a theoretical basis for preventing abdominal fat deposition in broiler chickens.

## MATERIALS AND METHODS

### Cell Culture

The ICP2 cells were obtained from key laboratory of animal genetics, breeding and reproduction at Northeast Agricultural University, which were cultured in DMEM/F12 complete medium (Gibco) supplemented with 10% FBS (BI, Israel), 1% penicillin and 1% streptomycin at 37 °C with 5% CO_2_. Upon reaching around 80% confluence, the cells were rinsed with PBS (Wuhan Servicebio technology Co., Ltd, Wuhan, China) and then treated with trypsin for 30 s for to facilitate passage culture.

### Butyric Acid Treatment Time and Concentration Selection During Cell Proliferation

Cells were seeded in 96-well plates at a density of 5 × 10^3^ cells/well and incubated for 12 h, then treated with DMEM/F12 complete medium containing 0 to 14 mM butyric acid (Shanghai aladdin Biochemical Technology Co., Ltd, Shanghai, China) at 37°C for 24h and 48h. Afterward, 10% CCK-8 solution (Mickey Mouse Biotechnology Co., Ltd, Xi'an, China) was added to each well and incubated at 37°C for 2 h. Finally, the absorbance was measured at 450 nm using microplate reader (SynergyHT, BioTek) to evaluate cell viability. Based on the results of CCK-8 analysis, cell was inoculated in 6-well plates with 6 replicates per group and incubated for 12 h. Subsequently, they were treated with DMEM/F12 complete medium containing 12 mM butyric acid at 37°C for 48h. Precipitation cells were collected to detect relevant indicators for subsequent analyses.

### Lipid Quantification by Oil Red O Staining

The oil red O storage solution (Xi'an Ao Rui Jing Chuang Biological Technology Co., Ltd, Xi'an, China) was diluted by mixing with ultra-pure water in a ratio of 3:2. After 30 min at room temperature, the mixture was filtered with 0.45μm filter to get Oil Red O working solution. The differentiated adipocytes underwent 3 washed with PBS before being fixed overnight with 4% formaldehyde (Wuhan Servicebio technology Co., Ltd, Wuhan, China). Following fixation, the cells were washed again and subsequently stained with oil red O working solution for 2 h at room temperature and observed with an inverted microscope after washing with PBS. Finally, the Oil Red O in cells was eluted with 100% isopropyl alcohol via incubating in a shaker for 15 min, and the absorbance at 510 nm was measured to determine the lipid content.

### Butyric Acid Treatment Time and Concentration Selection During Cell Differentiation

ICP2 cell lines were inoculated in 6-well plates and the DMEM/F12 complete medium was replaced daily. When cells growth reached approximately 80%, differentiation was induced by the DMEM/F12 differentiation medium containing 10% FBS, 1% penicillin, 1% streptomycin and 200 μM oleic acid (Xi'an Kunchuang Biotechnology Co., Ltd, Chain), alongside treatment with 0 to 8 mM butyric acid for 3 d, the lipid content was detected by quantification of oil red O staining to chosen the treatment concentration of butyric acid. Based on the results of oil red O staining, cells were inoculated in 6-well plates with 6 replicates per group. Subsequently, they were treated with DMEM/F12 differentiation medium containing 200 μM oleic acid and 4 mM butyric acid for 3 d. Precipitation cells were collected to detect relevant indicators for subsequent analyses.

### Determination of TC and TG Contents in Adipocytes

200 μL PBS was added to the cell precipitation and homogenized at 60 Hz for 30 s, followed by freezing at -80°C for 20 min. After thawing at room temperature, it was homogenized again at 60 Hz for 30 s. The cell suspension was prepared by repeating these steps 3 times. The protein levels were measured with BCA protein assay kit (Xi'an AccuRef Scientific Co., Ltd, Xi'an, China). Furthermore, total cholesterol (**TC**) and triglyceride (**TG**) levels were measured by a commercial kit (Nanjing Jiancheng Bioengineering Institute, Nanjing, China).

### Transcriptomics Analysis

The total RNA from cells of each group were extracted using Trizol reagent (Accurate Biotechnology Co., Ltd, Xi'an, China), followed by preparation of cDNA libraries for qualified RNA using theIllumina TruSeq Kit (Illumina, San Diego). Thereafter, sequencing was performed on the Illumina Hiseq platform by Shanghai Personal Biotechnology Co., Ltd. The raw data was filtered, quality controlled, and then aligned to the chicken genome (GRCg7b, https://www.ncbi.nlm.nih.gov/genome/111v) by HISAT2 software (http://ccb.jhu.edu/software/hisat2/index.shtml). Subsequently, the reads aligned to each unigene in each sample were quantified and converted to FPKM values to determine gene expression levels. Differential expression genes (**DEG**) were identified according to a threshold of *P*-value < 0.05 and log2 (fold change) ≥ 2. Next, R software was used for clustering heat map and PCA analysis. Gene Set Enrichment Analysis (**GSEA**) was performed using cluster profiler, and the significant enrichment criterion was *P < 0.05*.

### qRT-PCR

The extracted RNA was reversed transcription into cDNA, and quantitative measurements were performed with 2×SYBR Green qPCR Master Mix (US Everbright Inc., Nanjing, China). Subsequently, real-time fluorescence quantitative PCR was performed (Bioer, Hangzhou, China). The reaction conditions involved predenaturation at 95 °C for 3 min, followed by 40 cycles of 95 °C for 10 s and 60 °C for 30s. The primer sequences were synthesized by Shenggong Bioengineering Co., Ltd as shown in Supplementary Table S1. Relative expression of the target gene was calculated through the 2^−△△Ct^ method with β-actin serving as the internal reference.

### Western Blot Analysis

The total protein of ICP2 cell was extracted using RIPA lysis buffer (Zhonghui Hecai Co., Ltd, Xi'an, China), and the protein concentration was determined with BCA protein assay kit. Equal amounts of protein were loaded on SDS-PAGE gel for electrophoresis, and then transferred into PVDF membranes (Solarbio Life Science Co. Ltd, Xi'an, China). Subsequently, the membranes were blocked with 5% BSA and incubated with the primary antibody overnight at 4°C, followed by incubating with secondary antibody for 90 min. Details of antibodies were shown in Supplementary Table S2. Finally, the quantified the blotting bands density with Image-Pro Plus (National Institutes of Health).

### Statistical Analysis

All data were statistically analyzed and plotted using GraphPad Prism 8 (San Diego). Results were presented as Mean ± SEM, and the comparisons among different groups were calculated using the unpaired Student's *t* test with SPSS 20.0 (Chicago). Statistical significance was determined at *P < 0.05*.

## RESULTS

### Effect of Butyric Acid on ICP2 Cells Growth

The effect of butyric acid on ICP2 cells growth were assessed using the CCK8 assays. The results were shown in Supplementary Figure S1, butyric acid addition (2, 4, 6, 8, 10, 12, and 14 mM) has no effect on ICP2 proliferation after 24 h treatment (*P >* 0.05), whereas more than 8 mM dosage of butyric acid decreased ICP2 proliferation after 48 h treatment (*P* < 0.05). Considering the effect of butyric acid dosage on cytotoxicity, 12 mM butyric acid was used in subsequent proliferation experiment.

### Transcriptomic Analysis for Butyric Acid Affecting ICP2 Proliferation

**Figure 1:**
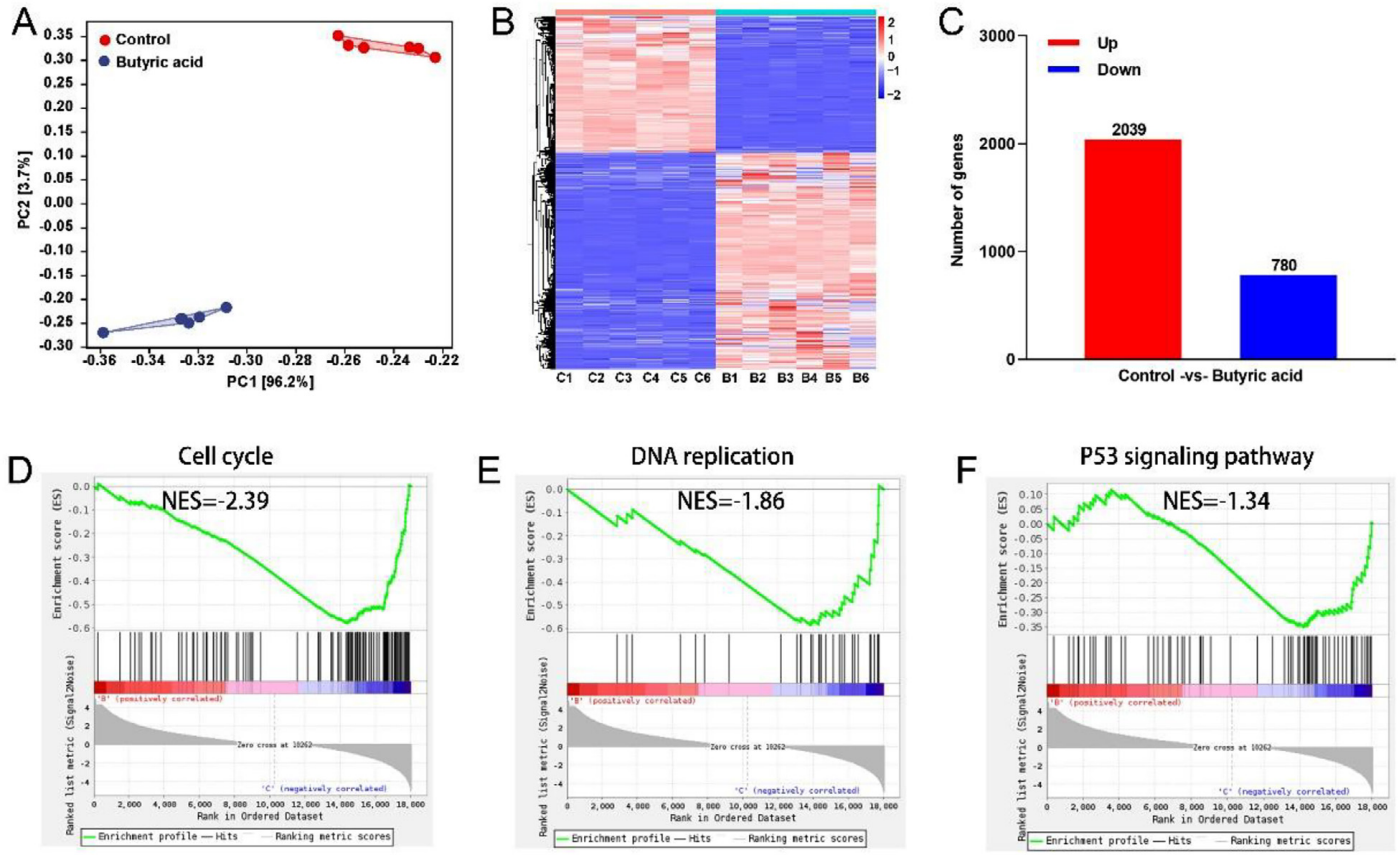
Transcriptomic analysis for butyric acid affecting ICP2 proliferation. (A) Principal components analysis (PCA) of Control and Butyric acid groups transcriptomes PC1 vs. PC2. (B) Cluster analysis heat map displayed up-regulated genes in red color, whereas down-regulated genes in blue color. (C) Statistical histogram of the number of up-regulated and down-regulated genes. (D–F) GSEA pathway enrichment from -Control and Butyric acid groups (n = 6).

To reveal transcriptional changes of butyric acid affecting ICP2 proliferation, RNA-seq was applied to identify DEGs. Principal component analysis (**PCA**) showed low similarity between Control and Butyric acid group (Figure 1A). DEGs clustering analysis was performed in the heat map where some genes elevated or reduced clearly in Butyric acid group (Figure 1B). Compared with the Control group, 2039 DEGs were significantly up-regulated and 780 DEGs were significantly down-regulated in Butyric acid group (Figure 1C). GSEA analysis indicated that Cell cycle, DNA replication and P53 signaling pathways were significantly enriched in Butyric acid group (Figures 1D–F).

### Butyric Acid Inhibited the Expression of Proliferation Marker Genes and Proteins

**Figure 2:**
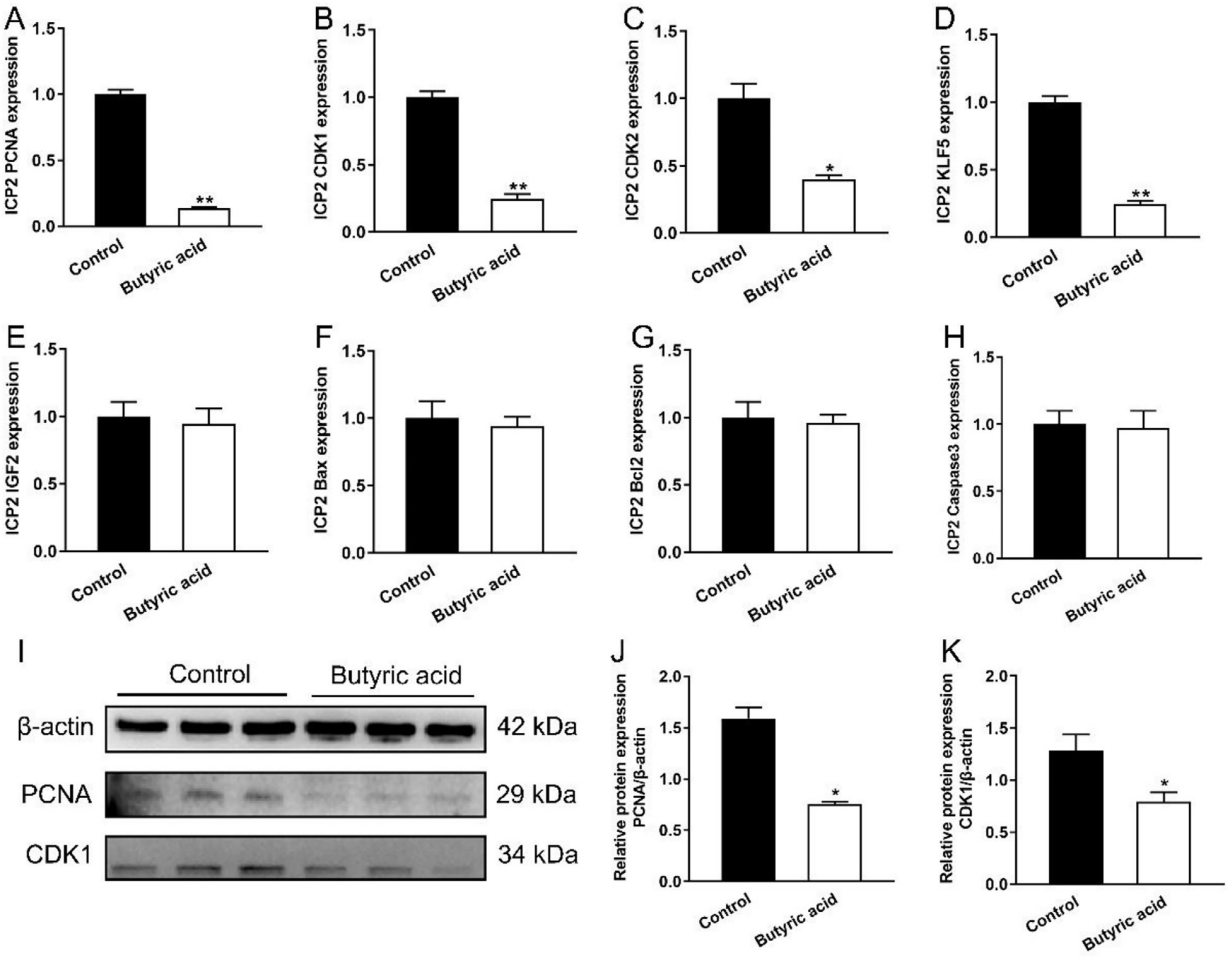
Butyric acid inhibited the expression of proliferation marker genes and protein in ICP2 cells. Gene expression of selected DEGs by RT-qPCR. Relative mRNA expression of PCNA, KLF5, CDK1, CDK2, ELOVL6, Bax, BCL2 and Caspase3 in ICP2 cells after treatment with 12mmol/L butyric acid for 48h (A–H, n = 6). Western blot analysis of protein expression associated with adipocyte proliferation (I). Quantitative analysis of Western blot bands which were normalized to β-actin (J-K, n = 3). Data were presented as means ± SEM.

To clarify the regulatory effect of butyric acid on ICP2 proliferation, we screened some proliferation marker genes from DEGs and confirmed their expression levels through qRT-PCR. The results indicated that the mRNA expression of PCNA, CDK1, CDK2 and KLF5 were significantly decreased in Butyric acid group (*P* < 0.05, Figures 2A–D). Correspondingly, the protein levels of PCNA and CDK1 were also reduced (*P* < 0.05, Figures 2G–K). Furthermore, we examined apoptotic-related factors, the results indicated that butyric acid had no effect on the expression of Bax, BCL2 and Caspase3 (*P* > 0.05, Figures 2F–H), which ruled out the possibility that the suppressive effect of butyric acid on cell growth was attributable to apoptosis.

### Butyric Acid Inhibits Lipid Droplet Production in ICP2

The effect of Butyric acid on ICP2 differentiation were observed through oil red O stainingin combination with absorbance measurement. Compared with the undifferentiated cells, ICP2 cells displayed lipid droplet formation following induction differentiation with 200 μM oleic acid for 1 d. With prolonged induction time, cells morphology exhibited more rounded, accompanied by a gradual increase in lipid droplet accumulation, and the quantitative colorimetric analysis of oil red O extraction revealed that the content of oil red O in ICP2 further increased with longer induction time (*P*<0.05, Supplementary Figure S2).

**Figure 3:**
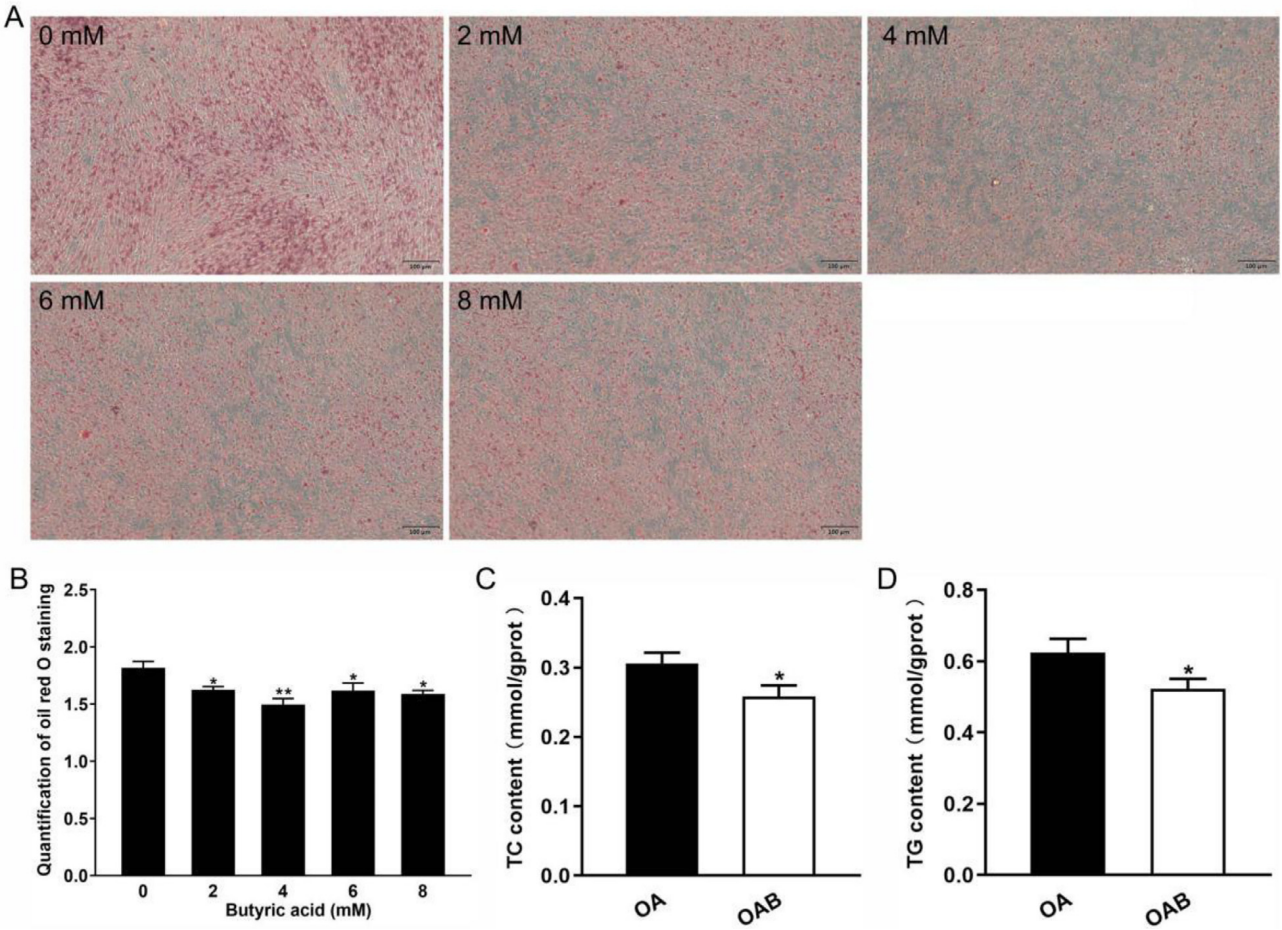
Butyric acid inhibited lipogenic of ICP2 cells. (A) Oil red O staining of differentiated adipocytes (magnification: 10×10; scale bar 100 um). (B) The semi-quantitative absorbance value of Oil Red. (C) TC and TG levels in adipocytes, and data were presented as means ± SEM (n = 8).

Considering the influence of butyric acid dosage on the toxicity and proliferation of ICP2 cells, the butyric acid concentrations ranging from 2 to 8 mM in differentiation experiments. As shown in Figures 3A–B, more than 2 mM butyric acid decreased the accumulation of red lipid droplets, with significant reduction in the semi-quantitative absorbance value of Oil Red (*P* < 0.05). Notably, the inhibitory effect of 4 mM butyric acid was highly significant (*P* < 0.01). Furthermore, 4mM butyric acid effectively inhibited both TC and TG levels of ICP2 (*P* < 0.05, Figure 3C).

### Transcriptomic Analysis for Butyric Acid Affecting ICP2 Differentiation

**Figure 4:**
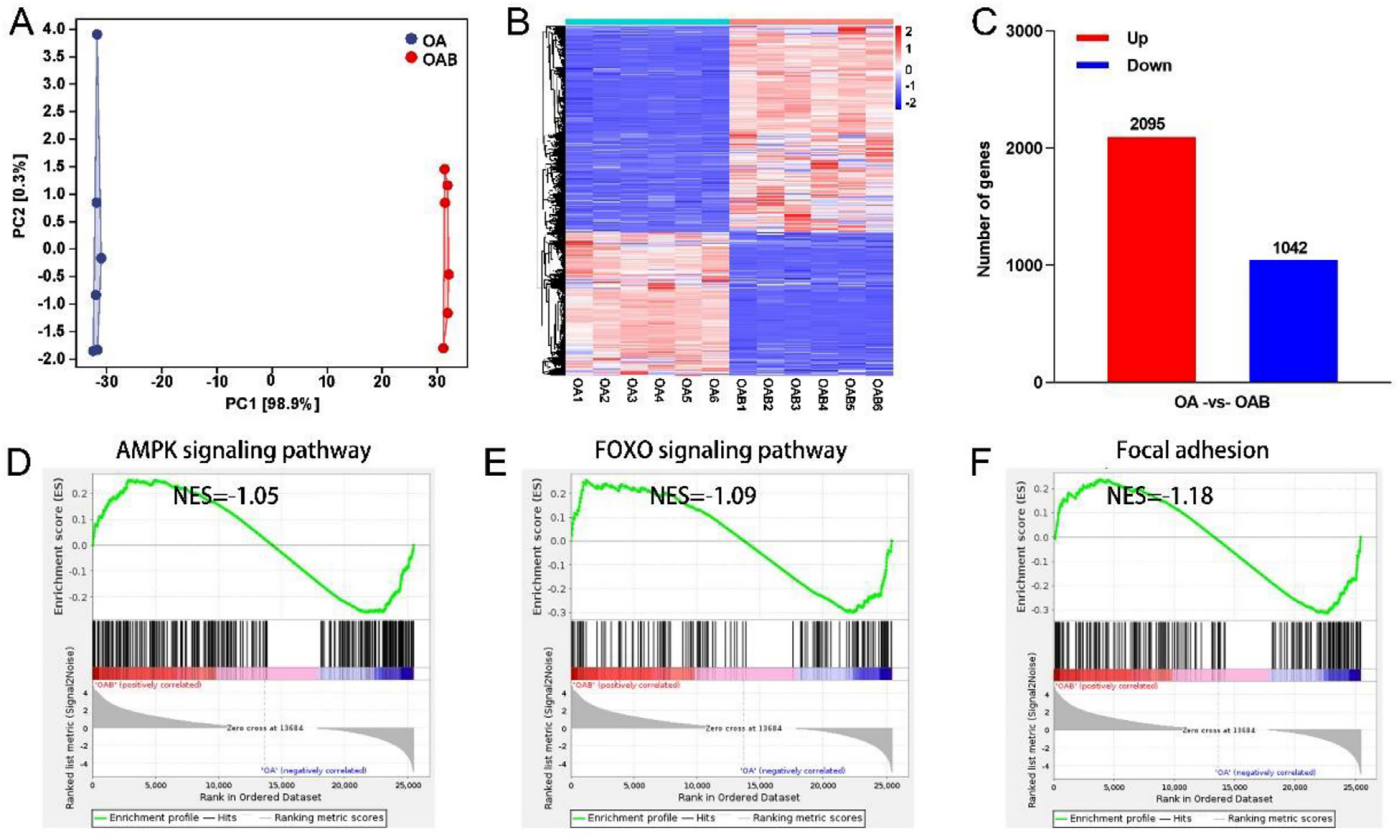
Transcriptomic analysis for butyric acid affecting ICP2 differentiation. (A) Principal components analysis (PCA) of OA and OAB groups transcriptomes PC1 vs. PC2. (B) Cluster analysis heat map displayed up-regulated genes in red color, whereas down-regulated genes in blue color. (C) Statistical histogram of the number of up-regulated and down-regulated genes. (D–E) GSEA pathway enrichment from OA vs. OAB (n = 6).

To reveal transcriptional changes during the ICP2 differentiation, RNA-seq was applied to identify DEGs. Principal component analysis (PCA) showed low similarity between OA and OAB group (Figure 4A). DEGs clustering analysis was performed in the heat map where some genes increased or decreased clearly in the OAB group (Figure 4B). Compared with the OA group, the number of up and down-regulated genes were 2095 and 1042 respectively in the OAB group (Figure 4C). GSEA analysis indicated that AMPK signaling pathway, FOXO signaling pathway and focal adhesion were significantly enriched in OAB group (Figures 4D–F).

### Butyric Acid Inhibited the Expression of Differentiation Marker Genes and Proteins

**Figure 5:**
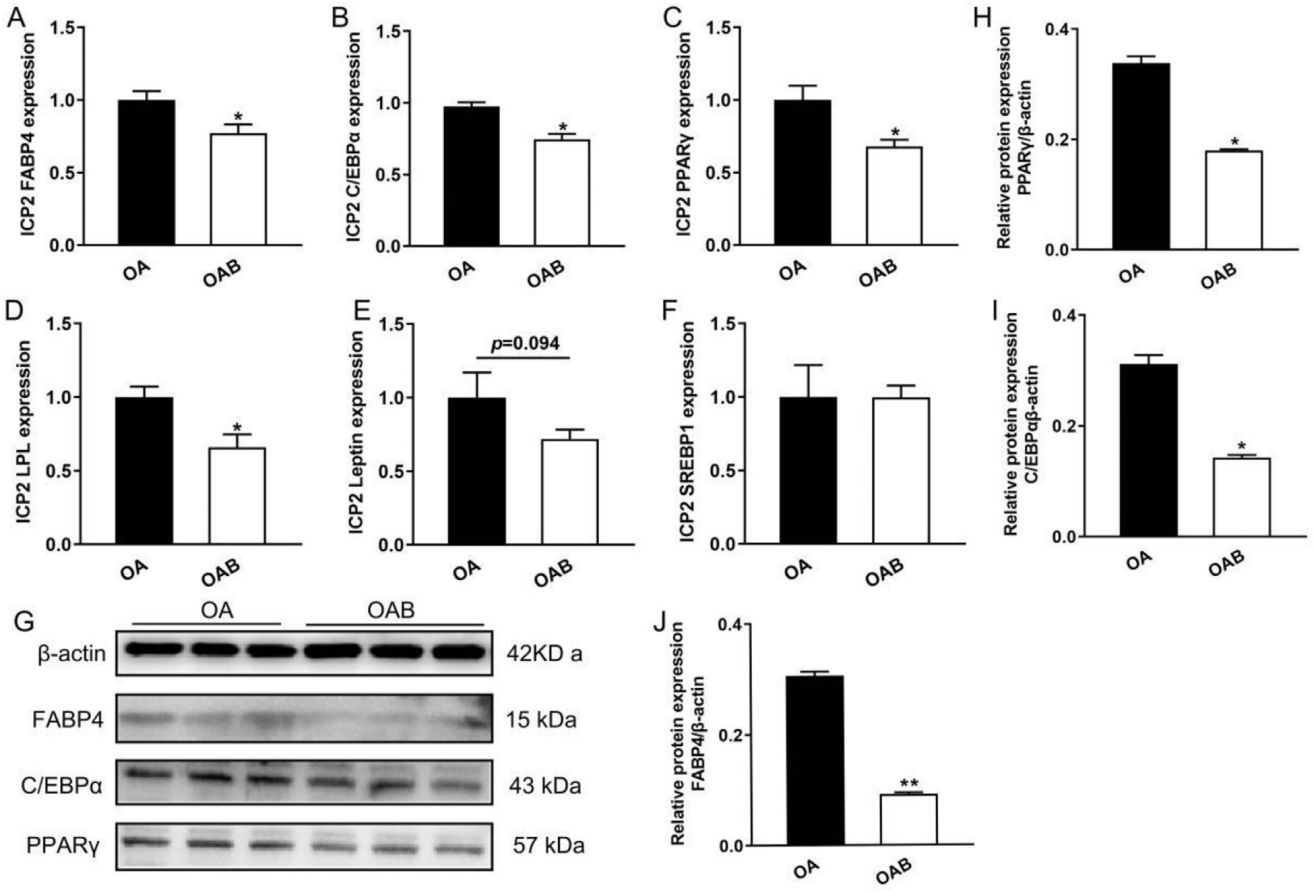
Butyric acid inhibited the expression of differentiation marker genes and protein in ICP2 cells. Gene expression of selected DEGs by RT-qPCR. Relative mRNA expression of FABP4, PPARγ, LPL, Leptin and SREBP1 in ICP2 cells (A-F, n = 6). Western blot analysis of protein expression associated with adipocyte differentiation (G). Quantitative analysis of Western blot bands which were normalized to β-actin (H-J, n = 3). Data were presented as means ± SEM.

To elucidate the regulatory effect of butyric acid on the differentiation of ICP2 cells, this study further investigated its effect on the expression of lipogenic marker genes. As depicted in Figure 5, the mRNA levels of representative genes associated with lipid differentiation, such as FABP4, C/EBPα, PPARγ and LPL were significantly down regulated in OAB group (*P* < 0.05, Figures 5A–D). Additionally, there was a decreasing trend in Leptin expression (*P* = 0.094, Figure 5E). But there was no significant effect on the expression of SREBP1 (*P >* 0.05, Figure 5F). Meanwhile, the protein levels of FABP4, C/EBPα and PPARγ were significantly alleviated in OAB group (*P* < 0.05, Figures 5H–J). These results suggested that butyric acid restrained lipid deposition of ICP2 cells by suppressing the expression of deposition marker genes.

## DISCUSSION

Fat deposition was due to the proliferation and differentiation of adipocyte. Adipocyte numbers increased due to preadipocyte proliferation, while adipocyte hypertrophy occurred when preadipocytes transform into mature adipocytes and accumulate triglycerides, causing an increase in their size and weight (An et al., 2023) (Nematbakhsh et al., 2021). SCFAs were metabolites produced by gut bacteria during the fermentation of dietary fiber and carbohydrates, which were not only an important energy source within body, but also served as signaling molecules to play a pivotal role in regulating energy metabolism (Fan et al., 2023). As one of the short-chain fatty acids, butyric acid could regulate lipid metabolism (Yu et al., 2019). Based on current research results of butyric acid on preadipocytes, different concentrations of butyric acid have different effects on the proliferation and differentiation of preadipocytes on different species. Specifically, butyric acid alleviated CIH-induced proliferation and lipid formation in human preadipocytes-subcutaneous through accumulation of human antigen R and inactivation of AMPK pathway (Su et al., 2021). Heimann found that 3 mM and 10 mM butyric acid reduced the lipolysis in a dose-dependent manner of primary rat adipocytes (Heimann et al., 2015). However, when mice fat cells were exposed to 0.2 mM, 2 mM and 20 mM butyric acid, it was observed that 2 mM and 20 mM significantly inhibited the synthesis of fatty acids and TG in the cells (Taggart et al., 2005). Hence, we proposed the hypothesis that butyric acid may regulate the proliferation and differentiation of ICP2, and intend to further investigate the influence of butyric acid on the proliferation and differentiation of ICP2 to clarify its role. This exploration will providing a theoretical basis for understanding the effects of butyric acid on body fat deposition in broiler chickens.

The proliferation and differentiation of preadipocytes were crucial for fat tissue formation (Van Nguyen et al., 2020). The rate and number of preadipocytes proliferation determined the degree of adipocyte development (Park et al., 2021), which further affected the fat percentage in the body and the occurrence of obesity (Ying and Simmons, 2021; Gao et al., 2017). Notably, transcription factors PCNA, CDK1, CDK2 and KLF5 assumed pivotal roles in preadipocyte proliferation by orchestrating critical mechanisms including cell cycle, DNA replication and P53 signaling pathway (Ioannilli et al., 2020; Yoo et al., 2006). PCNA was a cell cycle protein that was involved in DNA synthesis and promoted cell division and proliferation by interacting with other proteins (Ji et al., 2015). CDK1 and CDK2 were combined with cyclin to form complexes that promoted cell cycle progression and adipocyte proliferation (Martinez-Santibañez and Lumeng, 2014; Matsushime et al., 1994). In this study, we discovered that 12 mM butyric acid reduced the expression of genes such as PCNA, CDK1, CDK2 and KLF5, while the signaling pathways involved in cell cycle, DNA replication, and P53 signaling pathway were enriched. This discovery supported previous research (Su et al., 2021), indicating that butyric acid inhibited the proliferation of ICP2, potentially availed to reduce lipid accumulation. These observations suggested the necessity for additional research how butyric acid affected the differentiation of ICP2.

The transcriptional activation of specific genes were closely related to adipogenesis during adipocyte differentiation (Kaestner et al., 1989; Martinez-Santibañez and Lumeng, 2014). Pivotal genes involved PPARγ, C/EBP-α, SREBP1 and LPL, which served as markers for adipocyte differentiation. PPARγ belong to the PPAR family, not only promotes the differentiation of preadipocytes into mature adipocytes, but it also closely related to the formation of lipids in mature adipocytes. C/EBP-α was a pivotal regulator in the later stages of adipocyte differentiation, with its expression notably upregulated through the activation of PPARγ (Yeh et al., 1995). Meanwhile, LPL transcription was interregulated by PPARγ and LPL gene promoter (Schoonjans et al., 1996). Our research found that the addition of butyric acid down-regulates the expression of PPARγ and LPL during the differentiation of ICP2. Based on this observation, we supposed that butyric acid inhibited the activity of PPARγ, consequently reduced the expression of C/EBP-α and enhanced the lipolysis of LPL, which leaded to a decrease in the level of TG within ICP2 cell, ultimately inhibiting lipid deposition. Furthermore, SREBP1 was another crucial unclear transcription factor that collaboratively regulated preadipocyte differentiation alongside PPARγ and C/EBPα, exhibiting heightened expression levels in fat tissue (Taniguchi et al., 2008a; Taniguchi et al., 2008b). Jiao has demonstrated that butyric acid diminished the mRNA transcription of SREBP1, consequently impeded lipogenesis and TC synthesis in mice (Jiao et al., 2021). However, our experiment results revealed that butyric acid no effect on the expression of SREBP1 in ICP2 cell, which might be due to differences in the dosage of butyric acid administered or cell types.

The lipid metabolism was governed by multiple pathways, encompassing the AMPK signaling pathway, FOXO signaling pathway and Wnt signaling pathway. These pathways interacted to form complex regulatory networks that finely maintained homeostasis of lipid metabolism (Abdalla et al., 2018). AMPK regulated lipid metabolism by modulating the expression of PPARγ and SREBP-1, which respectively mediated the expression of lipolysis and lipoblast genes (Zou et al., 2021). Activation of the FOXO signaling pathway suppressed the expression of mitochondrial respiratory chain complexes and oxidative phosphorylation, thereby increased fatty acid oxidation and combated metabolic diseases such as obesity. Additionally, FOXO interacted with binding sites on PPARγ promoters to inhibit the transcriptional activity of PPARγ, thereby reduced fat deposition (Ioannilli et al., 2020). The study indicated that butyric acid alleviated CIH-induced proliferation and lipid formation in human preadipocytes-subcutaneous through accumulation of human antigen R and inactivation of AMPK pathway (Su et al., 2021). Consistent with the above results, our transcriptome analysis revealed that the addition of butyric acid during ICP2 differentiation significantly down-regulated AMPK signaling pathway and FOXO signaling pathway, and inhibited the expression of PPARγ, CEBP-α and FABP4. These results demonstrated that butyric acid as a signaling molecule activating AMPK signaling pathways and FOXO signaling pathways to regulate the expression of transcription factors associated with lipid metabolism, thereby indirectly regulated the synthesis of TG and ultimately inhibited lipid deposition during ICP2 differentiation.

## CONCLUSION

In summary, our study demonstrated that 12 mM butyric acid suppressed the proliferation of ICP2 cells by decelerating cell cycle progression, while 4 mM butyric acid impeded the differentiation of ICP2 cells by diminishing the synthesis of lipid droplets within the cytoplasm and inhibiting the expression of genes and proteins linked to lipid biosynthesis. These findings have provided a foundational understanding for further research into the impact of butyric acid on body fat accumulation of broiler chickens.

## DISCLOSURES

The author affirmed that all data in this study are freely accessible without limitation, and declared that they have no competing financial interests or personal relationships in this paper.

## Appendix Supplementary materials

Image, application 1

Image, application 2

## Footnotes

Supplementary material associated with this article can be found in the online version at doi:10.1016/j.psj.2024.104171.
