# Supplementary material for: Butyric acid reduced lipid deposition in immortalized chicken preadipocyte by inhibiting cell proliferation and differentiation
Source: Poult Sci. 2024 Aug 5;103(11):104171. doi: 10.1016/j.psj.2024.104171 (PMC11375136; doi:10.1016/j.psj.2024.104171)
Supplement: Supplementary file 2 [file mmc2.docx]

**Supplemental Table**

Table S1 Forward and reverse primer sequences for qRT-PCR analysis

| Gene | Accession number | Primer sequences, 5' to 3' | Product size, bp |
| --- | --- | --- | --- |
| β-actin | L08165 | F: ATTGTCCACCGCAAATGCTTC | 113 |
|  |  | R: AAATAAAGCCATGCCAATCTCGTC |  |
| PCNA | NM_204170 | F: CTGAGGGCTTCGACACCTAC | 142 |
|  |  | R: AGAGCCAACGTATCCGCATT |  |
| KLF5 | XM_040657735 | F: AAAAGACGCATCCACTAC | 196 |
|  |  | R: AACAGCCTCGGCAACAA |  |
| CDK1 | [NM_205314](https://www.ncbi.nlm.nih.gov/entrez/viewer.fcgi?db=nucleotide&id=2099356239) | F: CTGAGGACTTCCCTGCGGC | 143 |
|  |  | R: CAACACCATAGGTACCTTCCCC |  |
| CDK2 | NM_001199857 | TTCCGTATCTTCCGCACGTT | 171 |
|  |  | GTAGTGCAGCATTTGAGCCAG |  |
| ELOVL6 | XM_046916529 | F: GGTGGTCGGCACCTAATGAA | 169 |
|  |  | R: TCTGGTCACACACTGACTGC |  |
| Bax | [XM_040693909](https://www.ncbi.nlm.nih.gov/entrez/viewer.fcgi?db=nucleotide&id=2201791473) | F: CCTGGGGGTGAATGAGAACA | 242 |
|  |  | R: GAGCTGAGCGCCAACAGAT |  |
| BCL2 | NM_001025304 | F: GAGAAGCGATGCGCGAAAG | 148 |
|  |  | R: CCCGGTTACTGCTGGACATT |  |
| Caspase3 | [XM_046915477](https://www.ncbi.nlm.nih.gov/entrez/viewer.fcgi?db=nucleotide&id=2201792407) | F: AGGTGGAGGAGCTCTCCTATG | 199 |
|  |  | R: CCTGAGCGTGGTCCATCTTT |  |
| FABP4 | NM_204290 | F: GCCTGACAAAATGTGCGACC | 130 |
|  |  | R: ATTAGGCTTGGCCACACCAG |  |
| PPARγ | NM_001001460 | F: CCAAGGCAGCGGCAAAATAA | 188 |
|  |  | R: GTGCCCATAAATGATGGCCTAA |  |
| LPL | NM_205282 | F: CCGATCCCGAAGCTGAGATG | 186 |
|  |  | R: ACATTCCTGTCACCGTCCAC |  |
| Leptin | KT_970642 | F: GAAAAACTACGGGCGGATGC | 166 |
|  |  | R: GGAATCGCCTCAATCGTTGC |  |

Table S2 Antibodies information for western blotting

| Proteins | Company | Source | Molecular size, KDa | Dilution ratio |
| --- | --- | --- | --- | --- |
| β-actin | PTMbio (PTM-5028) | rabbit | 42 | 1:1000 |
| CDK1 | Abways (CY5176) | rabbit | 34 | 1:1000 |
| PCNA | Abways (AB0051) | rabbit | 29 | 1:1000 |
| FABP4 | Abways (CY6768) | rabbit | 15 | 1:1000 |
| C/EBPα | Abways (CY5723) | rabbit | 43 | 1:1000 |
| PPARγ | Abways (CY6675) | rabbit | 57 | 1:1000 |

Figure S1


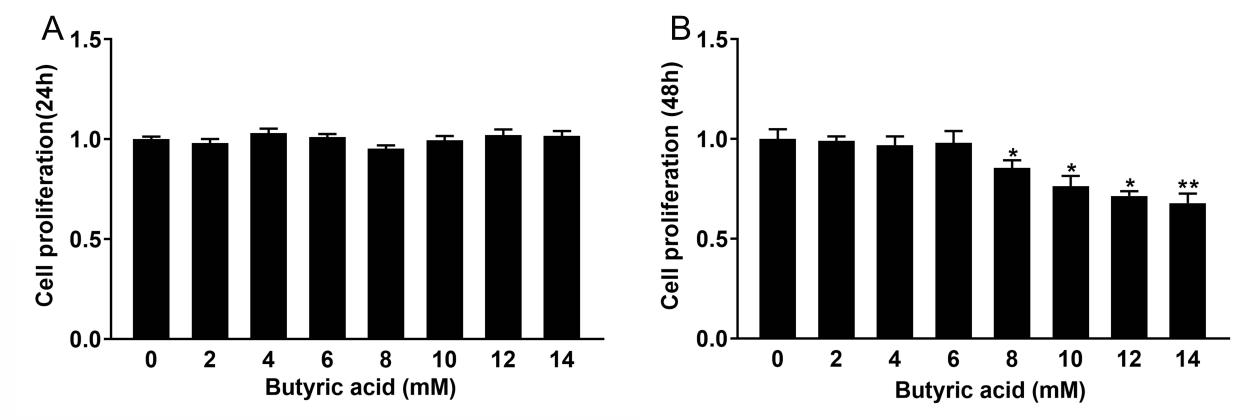


Exploring optimal concentration of butyric acid influencing the proliferation of ICP2 cells. The proliferation capacity of ICP2 cells was detected by CCK8 assay following treatment with butyric acid for 24h (A) and 48h (B). The asterisk (*) indicated statistically significant differences (two-tailed unpaired test, **P* < 0.05, ***P* < 0.01), the same below. Data were presented as mean ± SEM (n=8).

Figure S2


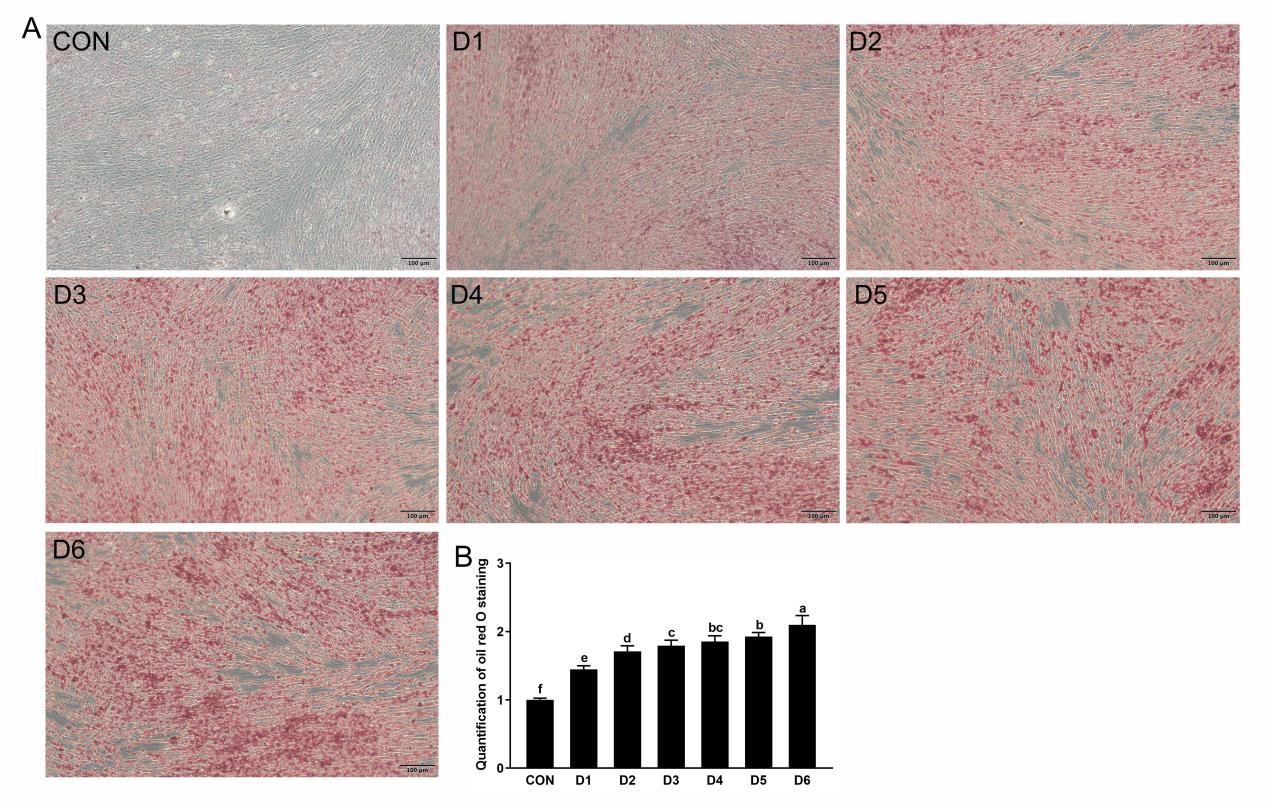


Oleic acid induced differentiation of ICP2 cells. (A) Oil red O staining of differentiated adipocytes (magnification: 10×10; scale bar 100 um). (B) The semi-quantitative absorbance value of Oil Red, and data were presented as means ± SEM (n=8).
